# Population-based estimates of engagement in HIV care and mortality using double-sampling methods following home-based counseling and testing in western Kenya

**DOI:** 10.1371/journal.pone.0223187

**Published:** 2019-10-02

**Authors:** Becky L. Genberg, Joseph W. Hogan, Yizhen Xu, Monicah Nyambura, Caren Tarus, Elyne Rotich, Catherine Kafu, Juddy Wachira, Suzanne Goodrich, Paula Braitstein

**Affiliations:** 1 Department of Epidemiology, Johns Hopkins Bloomberg School of Public Health, Baltimore, Maryland, United States of America; 2 Department of Health Services, Policy & Practice, Brown University School of Public Health, Brown University, Providence, Rhode Island, United States of America; 3 Department of Biostatistics, Brown University School of Public Health, Brown University, Providence, Rhode Island, United States of America; 4 Academic Model Providing Access to Healthcare (AMPATH), Eldoret, Kenya; 5 College of Health Sciences, School of Medicine, Moi University, Eldoret, Kenya; 6 Division of Infectious Diseases, School of Medicine, Indiana University, Indianapolis, Indiana, United States of America; 7 Epidemiology Division, Office of Global Public Health Education & Training, Dalla Lana School of Public Health, University of Toronto, Toronto, Ontario, Canada; KEMRI Wellcome Trust Research Programme, KENYA

## Abstract

**Introduction:**

Data on engagement in HIV care from population-based samples in sub-Saharan Africa are limited. The objective of this study was to use double-sampling methods to estimate linkage to HIV care, ART initiation, and mortality among all adults diagnosed with HIV by a comprehensive home-based counseling and testing (HBCT) program in western Kenya.

**Methods:**

HBCT was conducted door-to-door from December 2009 to April 2011 in three sub-counties of western Kenya by AMPATH (Academic Model Providing Access to Healthcare). For those identified as HIV-positive, data were merged with electronic medical records to determine engagement with HIV care. A randomly-drawn follow-up sample of 120 adults identified via HBCT who had not linked to care as of June 2015 in Bunyala sub-county were visited by trained fieldworkers to ascertain HIV care engagement and vital status. Double-sampled data were used to generate, via multinomial regression, predicted probabilities of engagement in care and mortality among those whose status could not be ascertained by matching with the electronic medical records in the three catchments.

**Results:**

Incorporating information from the double-sampling yielded estimates of prospective linkage to HIV care that ranged from 40–45%. Mortality estimates of those who did not engage in care following HBCT ranged from 12–16%. Among those who linked to care following HBCT, between 72–81% initiated ART.

**Discussion:**

In settings without universal national identifiers, rates of linkage to care from community-based programs may be subject to substantial underestimation. Follow-up samples of those with missing information can be used to partially correct this bias, as has been demonstrated previously for mortality among those who were lost-to-care programs. There is a need for harmonized data systems across health systems and programs.

## Introduction

In order to evaluate progress towards the UNAIDS goals of 90-90-90 [1,2], it is necessary to generate accurate estimates of engagement in the HIV care cascade [3], accounting for the losses occurring between each of these critical steps [4]. While facility-based estimates may be important for monitoring progress toward viral suppression for those who access care, population-based data are necessary to determine the impact of ART at the individual and population levels [5]. In particular, measuring the “second 90”–the initiation of ART among all those diagnosed with HIV–requires beginning the cascade at the point of HIV diagnosis, rather than enrollment in care, as has been seen in most studies [6]. Despite the global focus on achieving the UNAIDS goals, however, data on linkage to care following HIV testing and counseling outside of healthcare facilities or randomized controlled trials in sub-Saharan Africa remains limited [7].

From existing research, we know that barriers to engaging in care following HIV diagnosis in resource-limited settings are multilevel, encompassing individual, social, and structural factors [8–10]. Yet little research has gone beyond characterizing those who do not link to care and the barriers to engaging in care. As a result, the outcomes among those who are diagnosed with HIV and never link to care remain unknown. Prior research demonstrates that a substantial proportion of those who enroll in care but are then counted as “lost-to-follow-up” from HIV treatment programs across sub-Saharan Africa are actually deceased [11,12]. It is therefore critical to gather additional data on those who are lost in the early phases of the HIV care continuum, prior to enrolling in care, in order to accurately estimate progress toward 90-90-90 and design interventions to address the needs of individuals who never engage with HIV care following diagnosis.

The objective of this study, therefore, was to employ double-sampling methods to estimate linkage to care and mortality. We focused on data regarding HIV diagnosis generated from home-based counseling and testing (HBCT) because it allowed us to make estimates of engagement in HIV care using a population-based sample of people living with HIV in one geographical region. This paper reports on the additional follow-up among adults who were identified as HIV-positive via HBCT in western Kenya and whose linkage to care status was unknown as of June 2015.

## Methods

### Study setting

This study was situated within the Academic Model Providing Access to Healthcare (AMPATH) HIV care and treatment program in western Kenya. AMPATH is a joint partnership between Moi University College of Health Sciences and Moi Teaching and Referral Hospital in Eldoret, Kenya, and a consortium of North American universities and has been described elsewhere [13,14]. AMPATH has enrolled over 150,000 people living with HIV in care and treatment since 2001 and conducted home-based counseling and testing (HBCT) for HIV from 2009–2015. This study was reviewed and approved by the Institutional Research & Ethics Committee at Moi University and the Research Ethics Board of the University of Toronto. Informed consent was obtained by participants prior to participation and documented by the fieldworker.

### Home-based counseling and testing (HBCT)

HBCT was offered door-to-door in Bunyala, Chulaimbo and Teso catchments in Kenya between December 2009 and April 2011. HBCT has been described elsewhere and reached approximately 85% of the population living in Bunyala [15]. Briefly, following community mobilization, counselors trained according to the Ministry of Health requirements and WHO guidelines systematically offered HIV testing door-to-door within AMPATH catchment areas. Individuals with known HIV diagnosis were offered repeat testing and referrals. Those diagnosed with HIV were offered referrals for care. Data were collected on household socio-demographic and economic characteristics, previous HIV testing history, and HIV testing uptake and results during the HBCT encounter.

### Sampling frame

**Fig 1:**
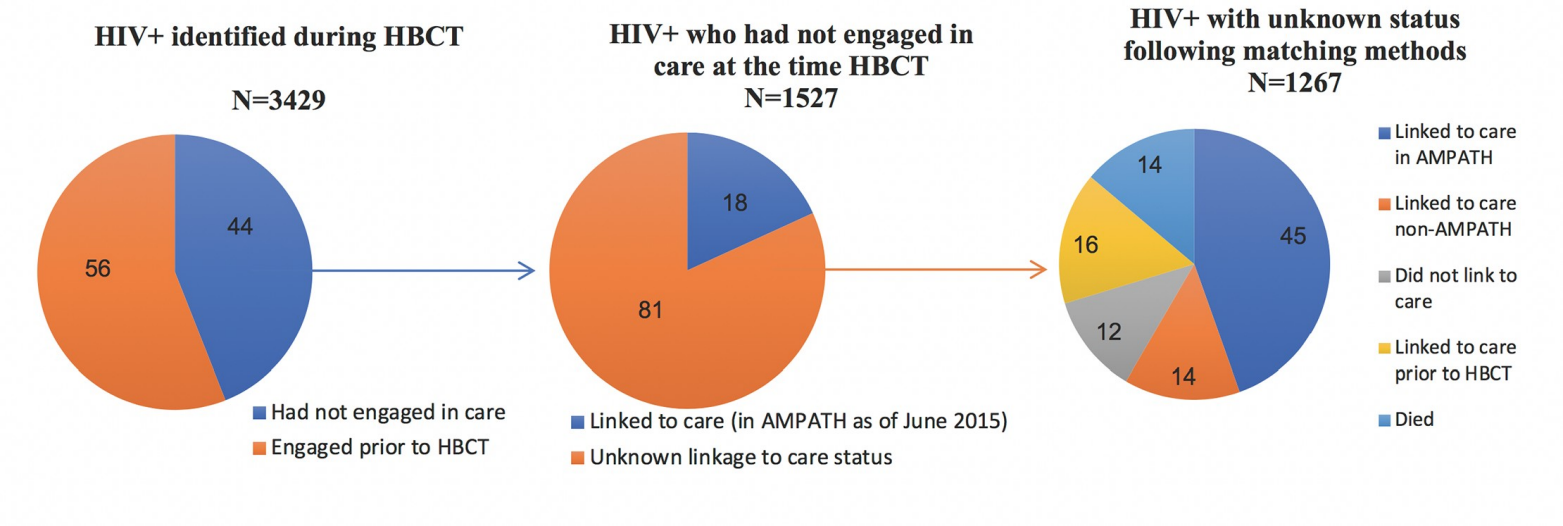
Pie charts summarizing engagement in care based on data matching methods at the time of HBCT (left), prospective linkage to care as of June 2015 (center) and updated estimates based on imputation models from double-sampled data (right).

The sampling frame for the study sample included individuals living in Bunyala who were identified as HIV-positive at the time of HBCT and whose linkage to care status was unknown following methods to determine engagement in care status (Fig 1).

We used two methods to determine engagement in care status following HBCT in Bunyala. First we used probabilistic matching to determine likely matches based on two or three names, dates of birth, and sex in the HBCT and AMPATH medical records (AMRS) as has been described previously [16]. Next we used deterministic matching to identify additional matches among the newly diagnosed within the Bunyala catchment [17]. For the purposes of this study, we assumed that having a record of an initial encounter with an HIV care provider within the AMRS indicated that the individual had engaged in HIV care at least once. The sampling frame included all individuals 18 years of age or older at the time of HBCT who were diagnosed with HIV in Bunyala sub-county of Kenya, and who had not linked to care according to AMPATH AMRS records as of June 2015.

### Double-sampling

A random sample of 120 individuals from the above sampling frame in Bunyala was drawn. We generated lists of the selected individuals and provided them to trained fieldworkers for further follow-up.

### Study procedures

Trained fieldworkers were assigned to visit the randomly selected individuals between July 2015 and June 2016 to ascertain additional data on: vital status, residence location, engagement in HIV care since HIV diagnosis, reasons for not engaging in care (if applicable), and point-of-care CD4 testing (if person was located). Last known residential status was obtained from data collected at the time of HIV testing.

Upon visiting the last known residential location of the selected individual, if the person was identified at the location, fieldworkers explained the purpose of the study, obtained informed consent, and conducted a brief interview to ascertain their engagement in HIV care. Those who reported being in care were asked to show their clinic enrollment card, whether for AMPATH or other care programs. Fieldworkers conducted a finger prick to ascertain CD4 status using the Alere CD4 test kit. Results were provided to individuals immediately and referrals were provided for those who had not engaged in HIV care.

If the fieldworker did not locate the selected individual at the last known residential location, additional information was sought from informants, including family members and/or neighbors, regarding the vital status of the selected individual without explaining the specific nature of their visit. If the individual was alive and had relocated within Bunyala, they visited the individual at their new residential location. If the selected individual was alive had relocated outside of the catchment, an attempt was made to make contact by phone. If they were unable to contact the selected individual by phone, no further information was available. If an informant reported that the selected participant was deceased, the date and cause of death was ascertained according to standard verbal autopsy methods. Deaths were confirmed where possible with a local registry kept by the Area Chief or sub-Chief.

For individuals who were found to have engaged in HIV care in AMPATH, data were extracted from the electronic medical record where possible. Data on demographic characteristics and previous HIV testing history were taken from HCT data collected at the time of HIV testing and diagnosis.

### Statistical analysis

Descriptive statistics were used to describe the socio-demographic characteristics of the sampling frame in Bunyala and the randomly selected sample. We also described the population of those with unknown care status from Chulaimbo and Teso following deterministic record matching methods in these two catchments. We compared the randomly selected sample to the overall population from Bunyala using t-test for age and chi-squared test for other categorical variables. For those who were located and reported not to have linked to care following HIV diagnosis, the frequencies of responses of reasons for not linking to care were also calculated.

For those selected who were identified for additional follow up, we defined the outcome to have five categories: linked to HIV care within AMPATH prior to HBCT, linked to HIV care within AMPATH following HBCT, linked to HIV care outside AMPATH, dead, and not engaged in care. Our goal was to draw inference about the probability of linkage to care and death, stratified by catchment. We calculated the linkage distribution among those missing linkage information (i.e., those who were not matched in AMRS or found via double-sampling and additional follow-up).

To achieve this goal, we statistically imputed the categorical outcome probabilities for those people with missing care status. Using the double-sampled data to estimate the outcomes of interest, a multinomial regression model was fitted to those with linkage outcome observed, using the following covariates: age, gender, number of children in household, previous-testing status, education status, marital status, amount of land and animals owned, household population, and occupation status. The model was used to generate predicted linkage status probabilities for those whose linkage status was missing. We used nonparametric bootstrap resampling (with 1000 bootstrap draws of the full dataset) to reflect both sampling variability and prediction variance in our estimates of uncertainty. For each bootstrapped sample, we fit a multinomial logit model based on those who were double sampled and had been located. We used the fitted model to estimate outcome probabilities for those with missing linkage status. Next, we calculated the linkage status probabilities for the entire bootstrapped sample for those with unknown linkage and the observed linkage estimates for those with observed linkage data. This yielded 1000 sets of linkage probability estimates. The bootstrap procedure yielded an overall estimate of linkage status probabilities and associated measures of uncertainty that are reflected in confidence intervals and histograms. For a specific bootstrapped sample, we averaged among each outcome category to obtain an estimated probability for the category, stratified by catchment. Finally, we further stratified by sex to examine differences in outcomes.

We conducted two sensitivity analysis. First we examined the impact of extreme cases of those whose status remained unknown following double-sampling on the estimates. Second, we examined the model excluding those whose linkage status preceded HBCT. All statistical analysis was completed using R 64bit version 3·2·5.

## Results

### Engagement in care and ART initiation according to record-matching methods

Table 1 presents estimates of engagement in care and ART initiation from the full sample of those identified as HIV-positive at the time of HBCT from the three catchment areas. Engagement in care prior to HBCT was 55%, 25%, and 38% in Bunyala, Chulaimbo, and Teso, respectively according to record matching methods. Among those who had engaged in care in AMPATH prior to HCT, 94%, 92%, and 87%, in Bunyala, Teso and Chulaimbo, respectively, had initiated ART as of June 2015. Estimates of linkage from this study using multiple data merge methods were 19% in Bunyala, 12% in Chulaimbo and 17% in Teso. Among those who linked to care in AMPATH following HBCT, 81%, 76%, and 72%, in Bunyala, Teso and Chulaimbo, respectively, initiated ART as of June 2015.

**Table 1:** Estimates for engagement in care at the time of HBCT, linkage to care and ART initiation as of June 2015 determined by matching methods, by catchment.

	Bunyala N = 3429	Chulaimbo N = 3913	Teso N = 869
Linked AMPATH before HCT, n (%)	1902 (55)	983 (25)	330 (38)
Initiated ART, n (%)	1779 (94)	851 (87)	304 (92)
Not engaged in care at time of HBCT, n (%)	1527 (45)	2930 (75)	539 (62)
Linked AMPATH following HCT, n (%)	284 (19)	340 (12)	94 (17)
Initiated ART, n (%)	230 (81)	244 (72)	71 (76)

### Socio-demographic characteristics of those not identified with record-matching methods

Table 2 presents the socio-demographic characteristics of those whose linkage to care status remained unknown following data matching methods. In Bunyala, nearly 60% of those who were identified as HIV-positive with unknown care status were female and the median age was 31 years. Just over half had a primary school education, with 34% reporting no education, and 12% reporting secondary or tertiary school. Nearly 60% were married at the time of HBCT, 21% were unemployed, 14% lived in households that owned land, and 11% lived in households that owned animals. Nearly half of the sample (49%) reported having previously tested for HIV at the time of HBCT. From those with unknown status in Bunyala, the random sample for additional follow-up was drawn and the distribution of socio-demographic characteristics of the n = 120 individuals who were randomly selected for additional follow-up were not different from the overall sample of those with unknown linkage status following record-matching methods in Bunyala.

**Table 2:** Socio-demographic characteristics and HIV testing history of those identified as HIV-positive during HBCT whose linkage to care status was unknown following matching methods, by catchment, and for the sub-sample of those randomly selected from Bunyala for additional follow-up.

	Bunyala	Randomly selected	Chulaimbo	Teso
N	1267	120	2590	445
Female, %	60	57	67	72
Median age, in years	31	32	35	34
Educational attainment, %				
None	34	31	31	24
Primary	53	56	55	57
Secondary/Tertiary	12	13	13	19
Marital status, %				
Single	17	10	11	18
Married/cohabitating	58	67	57	54
Divorced/separated	10	9	6	14
Widowed	15	14	26	13
Unemployed, %	21	18	21	17
Household owns any land, %	14	14	23	24
Household owns any animals, %	11	8	23	19
Previously tested for HIV, %	49	26	73	58

In Chulaimbo and Teso, there was a slightly higher proportion female (67% and 72%, respectively), compared to Bunyala (60%, p = 0·54) and a higher median age (35 and 34 years, respectively), compared to Bunyala (31 years, p<0·01). Educational attainment, marital status and unemployment were similar to Bunyala, however, those in Chulaimbo and Teso had slightly higher proportion owning land (23% and 24%, respectively) compared to 14% in Bunyala (p = 0.18), and a higher proportion animals (23% and 19%, respectively), compared to 11% in Bunyala (p<0·05). A higher proportion of the sample in Chulaimbo and Teso also reported having previously tested for HIV (73% and 58%, respectively), compared to Bunyala (49%, p<0·01).

### Double-sampling outcomes

Among the 120 who were selected for additional follow-up, 33 could not be located and no further information was available. The data from these individuals was therefore retained in the sample of those with unknown care status. Eighteen of the randomly selected were found to have engaged in care prior to HBCT. For the remaining 69 individuals, information was collected regarding their vital status and engagement in HIV care. Of those 69, 39 reported engaging in HIV care since being diagnosed via HBCT, 24 within AMPATH and 15 outside of AMPATH. Sixteen individuals reported never having engaged with HIV care since the time of their diagnosis. Finally, 14 individuals had died since HBCT. Based on the estimated time of death, the median time from diagnosis via HBCT and death was 2·1 years.

Table 3 presents the estimates of linkage to care following HBCT according to the imputation model for those with unknown status, stratified by sex and catchment, with density probability curves of the five outcomes by catchment depicted in S1 Fig.

**Table 3:** Estimated proportion of linkage to HIV care derived from imputation models, within and outside of AMPATH, and mortality, among those identified as HIV-positive during HBCT whose care status was unknown following record-matching, by sex and catchment.

	Bunyala			Chulaimbo			Teso		
	Female (n = 712)	Male (n = 468)	Total (n = 1180)	Female (n = 1746)	Male (n = 844)	Total (n = 2590)	Female (n = 318)	Male (n = 127)	Total (n = 445)
Linked before HBCT	13 (5, 21)	21 (12, 30)	16 (10, 23)	15 (5, 25)	20 (8, 34)	17 (8, 26)	14 (6, 23)	19 (8, 31)	15 (8, 24)
Linked to HIV care (in AMPATH)	50 (37, 61)	39 (29, 50)	45 (35, 54)	45 (32, 58)	31 (19, 46)	40 (29, 53)	48 (34, 60)	38 (28, 50)	45 (33, 55)
Linked to care outside of AMPATH	13 (5, 23)	15 (7, 26)	14 (7, 23)	17 (5, 31)	21 (7, 37)	18 (6, 31)	16 (6, 29)	18 (6, 34)	17 (7, 29)
Not linked to HIV care since HBCT	13 (6, 22)	10 (4, 18)	12 (6, 19)	9 (3, 19)	8 (3, 18)	9 (4, 19)	11 (4, 22)	10 (3, 20)	11 (4, 20)
Died since HBCT	12 (6, 20)	15 (7, 24)	14 (7, 20)	14 (5, 24)	19 (6, 32)	16 (6, 25)	11 (4, 20)	14 (4, 26)	12 (5, 20)

Estimates may exceed 100% due to rounding.

Estimates of linkage to care within AMPATH based on the imputed probabilities were 45% (35–54), 40% (29–53), and 45% (33–55) for Bunyala, Chulaimbo, and Teso, respectively. Estimates of other outcomes included: 16% (10–23), 17% (8–26), and 15% (8–24) of those with unknown care status in Bunyala, Chulaimbo and Teso, respectively, were found to have accessed care for HIV prior to HBCT; 14% (7–23), 18% (6–31), and 17% (7–29) linked to care outside of AMPATH, 12% (6–19), 9% (4–19), and 11% (4–20) had not linked to care since HBCT, and 14% (7–20), 16% (6–25), and 12% (5–20) had died in Bunyala, Chulaimbo, and Teso, respectively.

Imputed estimates demonstrated higher linkage to care among females across all three catchments (p<0·001). In addition, females had substantially lower mortality rates in each of the three sub-counties (p<0·001). Median CD4 counts at the time of double-sampling did not differ between those who had linked to care within or outside of AMPATH (median: 476 cells/mm^3^) and had not linked to care (median: 406 cells/mm^3^) since their diagnosis (p = 0·30). S1 Table includes coefficients from adjusted models. Additional sensitivity analysis examining extreme cases and excluding those who linked to care prior to HBCT are also included (S2 and S3 Tables).

### Reasons for not linking to care

The 15 individuals who were found not to have linked to HIV care since their diagnosis via HBCT cited the following main reasons for not engaging in care: conflicts with work (42%), stigma (40%), lack of resources for transport to the clinic (27%), and perceptions that they were healthy and felt well (27%). Reasons related to stigma included not wanting to be seen at the clinic, fears of being recognized and labeled as having HIV, and fears of unintended disclosure as a result of accessing care.

## Discussion

This study demonstrates that a higher proportion of individuals identified as HIV-positive in western Kenya linked to care following home-based counseling and testing than was previously reported using probabilistic matching methods only [16]. Our results across three catchments demonstrate that nearly half of those whose linkage to care status was unknown following data matching had in fact linked to care. Unfortunately, the data also demonstrated high mortality rates, with between 12–19% mortality among those who were identified as HIV-positive who had not engaged in care prior to HBCT and whose linkage to care status remained unknown following data matching methods.

There are limited data on mortality for those who do not access care following community-based HIV testing and diagnosis. Studies on mortality for pre-ART patients in sub-Saharan Africa demonstrate mortality rates between 15–25% [7]. While these rates are slightly higher than what we found among those identified as HIV-positive through HBCT, patients who access care and are subsequently lost may be different from those who never linked to care. Similar estimates of mortality to what we found was observed in the Siziani trial in South Africa, with 13% mortality at 12-months post-diagnosis, however, it was conducted among individuals presenting for HIV testing at a facility, another group that may differ from those who were diagnosed via home-based counseling and testing [18]. Taken together, our estimates suggest that a high proportion of individuals who are identified as HIV-positive through HBCT never engage in care and die within a median of two years following testing.

Estimates of linkage to care presented here are higher than previously reported [16] due to variation in the methods of estimation. Estimates of linkage to care based on merged administrative data may be underestimating the true linkage to care rates, particularly in settings that lack universal national identifiers and rely on imprecise dates of birth for vital statistics and care program registries. Our findings imply that additional resources are urgently needed to bolster health information systems globally in order to enhance the harmonization of data between programs and enable analysis of “big data” to produce accurate and reliable population-based health estimates.

Our findings suggest that the losses and deaths that occur prior to engagement in HIV care may be substantial. In order to achieve the “second 90” of the UNAIDS goals, additional effort will be needed to bolster support for linkage to care and initiation of ART among those who have never engaged with care, particularly outside of health facilities. In our study, those who never engaged in care was a combination of those who were newly diagnosed and those with a previously known diagnosis who had never accessed care. A recent systematic review demonstrated that linkage rates following home-based counseling and testing were higher when followed by additional strategies to engage those diagnosed with HIV in care beyond referral only [19]. Different strategies may be implicated for those who have repeatedly tested for HIV without engaging in care compared with those who are newly diagnosed. This may be particularly relevant in the era of universal ART when many of those newly diagnosed in community and home-based settings are not symptomatic.

There are several limitations of the current study. We conducted double-sampling with a small (10%) randomly drawn sample in Bunyala only due to resource constraints and inferred information from Bunyala to the two other sub-counties. This has two important implications. First, there may be differences in the relationships between the covariates and the outcomes by catchment. This analysis assumes a consistent relationship between covariates and outcomes within each of the three catchments of western Kenya. An implication of this assumption is that dissimilarity in linkage distribution across catchments can be explained by differences in covariate distributions. Secondly, the data from the additional outreach in Bunyala may not capture the distribution of outcomes among those who did not link to care in Chulaimbo and Teso. In particular there may be different availability of other treatment programs, beyond AMPATH, in the three regions, and receipt of care outside of AMPATH in Bunyala may not reflect the same in Chulaimbo and Teso.

Despite these limitations, this study is among the first to evaluate the outcomes of individuals who were identified as HIV-positive through HBCT in western Kenya who had not engaged in care. This study demonstrates that with additional follow-up data, more accurate estimates of engagement in care can be achieved compared to those calculated using data merging methods. We also demonstrated a high mortality rate for those who never linked to care, demonstrating the urgent need for interventions designed to speed access to care and ART among those diagnosed who have never engaged with care and those newly diagnosed with HIV in the community.

## Supporting information

S1 Table Adjusted multinomial logit coefficients for outcomes (linked to care outside of AMPATH, linked to care within AMPATH, linked to care before home-based counseling and testing (HBCT), death) compared to not linked to care among n = 87 individuals who were double-sampled and located in Bunyala. (DOCX)

S2 Table Estimated proportion of linkage to HIV care under sensitivity analysis, derived from imputation models, within and outside of AMPATH, and mortality, among those identified as HIV-positive during HBCT whose care status was unknown following record-matching, by sex and catchment. (DOCX)

S3 Table Estimated proportion of linkage to HIV care since HBCT derived from imputation models, within and outside of AMPATH, and mortality, among those identified as HIV-positive during HBCT whose care status was unknown following record-matching, by sex and catchment. (DOCX)

S1 Fig Density probability curves for linkage to care (in and outside of AMPATH), death and not linked to care, by catchment, among those with unknown linkage status. (TIFF)
