# Supplementary material for: Population-based estimates of engagement in HIV care and mortality using double-sampling methods following home-based counseling and testing in western Kenya
Source: PLoS One. 2019 Oct 2;14(10):e0223187. doi: 10.1371/journal.pone.0223187 (PMC6774575; doi:10.1371/journal.pone.0223187)
Supplement: S1 Table — (DOCX) [file pone.0223187.s001.docx]

**S1 Table. Adjusted multinomial logit coefficients for outcomes (linked to care outside of AMPATH, linked to care within AMPATH, linked to care before home-based counseling and testing (HBCT), death) compared to not linked to care among n=87 individuals who were double-sampled and located in Bunyala.**

|  | **Linked to care outside of AMPATH**  **(n=15)** | **Linked to care within AMPATH**  **(n=24)** | **Linked to care within AMPATH before HBCT (n=18)** | **Dead**  **(n=14)** |
| --- | --- | --- | --- | --- |
| Intercept | 0.41 (2.79) | 2.3 (2.52) | -22.53* (1.83) | -19.85* (1.76) |
| Age at HBCT | -0.01 (0.06) | -0.04 (0.05) | 0.04 (0.05) | 0.05 (0.05) |
| Sex (female) | 0.98 (1.02) | 0 (0.87) | 1.08 (1.06) | 1.41 (1.08) |
| Any children living in the household | 0.41 (0.88) | -0.41 (0.75) | -1.92* (0.92) | 0.36 (1.00) |
| Number of people in the household | -0.56 (0.82) | 0.62 (0.71) | 1.87* (0.85) | -0.61 (0.95) |
| Previously tested for HIV | 2.14 (1.41) | 2.25 (1.23) | 1.62 (1.34) | 2.56 (1.37) |
| Educational attainment  None (reference)  Primary education | 0.52 (1.10) | -1.08 (0.87) | 0.65 (1.05) | -0.92 (0.98) |
| More than primary education | -1.79 (2.40) | -0.8 (1.31) | 0.48 (1.40) | -1.58 (1.58) |
| Marital status  Single (reference)  Married/Cohabiting | -0.72 (1.70) | -2.02 (1.70) | 16.35* (1.05) | 18.13* (1.11) |
| Separated/Divorced/Widowed | 0.1 (1.70) | -1.29 (1.66) | 17.23* (1.21) | 18.62* (1.08) |
| Household owns any land | 1.59* (0.76) | -1.44 (1.11) | -1.05 (1.10) | 1.31 (0.88) |
| Number of animals owned by the household | 0.48 (0.34) | 0.02 (0.38) | -1.39 (0.99) | -13.73* (0.00) |
| Employment  Farmer/self-employed (reference)  Other (unemployed, informal worker, etc) | -1.13 (1.05) | 0.13 (0.75) | 0.61 (0.86) | 1.05 (1.01) |

Reference level: Not linked anywhere.

*: significance of coefficients from 2-tailed Wald z tests at $\alpha=0.05$
