# Supplementary material for: Population-based estimates of engagement in HIV care and mortality using double-sampling methods following home-based counseling and testing in western Kenya
Source: PLoS One. 2019 Oct 2;14(10):e0223187. doi: 10.1371/journal.pone.0223187 (PMC6774575; doi:10.1371/journal.pone.0223187)
Supplement: S2 Table — (DOCX) [file pone.0223187.s002.docx]

**S2 Table. Estimated proportion of linkage to HIV care under sensitivity analysis, derived from imputation models, within and outside of AMPATH, and mortality, among those identified as HIV-positive during HBCT whose care status was unknown following record-matching, by sex and catchment.**

|  | **Linked to care within AMPATH before HBCT** | **Linked to care within AMPATH after HBCT** | **Linked to care outside of AMPATH** | **Not linked to care** | **Dead** |
| --- | --- | --- | --- | --- | --- |
| Missing | 0.159 | 0.438 | 0.173 | 0.08 | 0.149 |
| Not linked to HIV care since HBCT | 0.145 | 0.338 | 0.139 | 0.283 | 0.096 |
| Linked to care outside of AMPATHH | 0.149 | 0.336 | 0.352 | 0.062 | 0.101 |
| Linked to HIV care (in AMPATH) | 0.144 | 0.55 | 0.147 | 0.064 | 0.096 |
| Died since HBCT | 0.138 | 0.348 | 0.151 | 0.063 | 0.300 |

Fixed level of the 33 unknown double sampled outcomes
