# Supplementary material for: Population-based estimates of engagement in HIV care and mortality using double-sampling methods following home-based counseling and testing in western Kenya
Source: PLoS One. 2019 Oct 2;14(10):e0223187. doi: 10.1371/journal.pone.0223187 (PMC6774575; doi:10.1371/journal.pone.0223187)
Supplement: S3 Table — (DOCX) [file pone.0223187.s003.docx]

**S3 Table. Estimated proportion of linkage to HIV care since HBCT derived from imputation models, within and outside of AMPATH, and mortality, among those identified as HIV-positive during HBCT whose care status was unknown following record-matching, by sex and catchment.**

|  | **Bunyala** | | | **Chulaimbo** | | | **Teso** | | |
| --- | --- | --- | --- | --- | --- | --- | --- | --- | --- |
|  | **Female**  **(n=712)** | **Male**  **(n=468)** | **Total**  **(n=1180)** | **Female**  **(n=1746)** | **Male**  **(n=844)** | **Total**  **(n=2590)** | **Female**  **(n=318)** | **Male**  **(n=127)** | **Total**  **(n=445)** |
| Linked to HIV care (in AMPATH) | 57 (43, 69) | 49 (36, 62) | 54 (42, 64) | 52 (38, 68) | 39 (23, 56) | 48 (34, 63) | 55 (39, 68) | 47 (34, 61) | 53 (40,64) |
| Linked to care outside of AMPATH | 15 (6, 26) | 19 (9, 31) | 16 (9, 27) | 20 (6, 36) | 27 (9, 45) | 22 (8, 37) | 19 (7, 33) | 23 (8, 41) | 20 (8, 33) |
| Not linked to HIV care since HBCT | 14 (7, 25) | 13 (5, 23) | 14 (8, 23) | 11 (4, 23) | 11 (3, 22) | 11 (5, 22) | 13 (5, 26) | 12 (4, 24) | 13 (5, 24) |
| Died since HBCT | 14 (7, 23) | 19 (9, 30) | 16 (9, 24) | 17 (6, 28) | 23 (8, 38) | 19 (8, 29) | 13 (5, 22) | 18 (6, 32) | 14 (6, 23) |

Estimates may exceed 100% due to rounding.
