# Supplementary figures and images for: Population-based estimates of engagement in HIV care and mortality using double-sampling methods following home-based counseling and testing in western Kenya
Source: PLoS One. 2019 Oct 2;14(10):e0223187. doi: 10.1371/journal.pone.0223187 (PMC6774575; doi:10.1371/journal.pone.0223187)

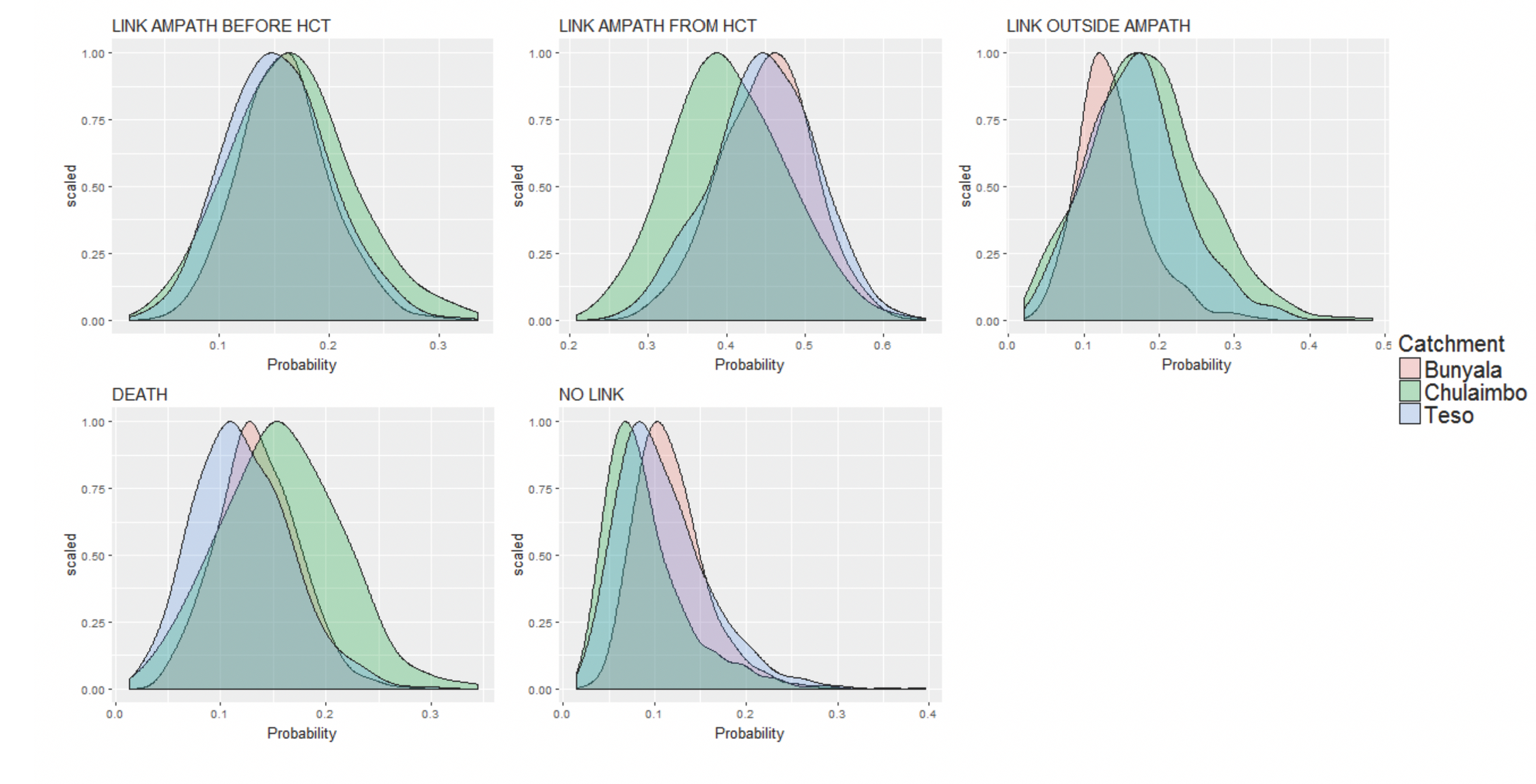

Supplement: S1 Fig — (TIFF) [file pone.0223187.s004.tiff]
